# Changes in serum interleukin-6 (IL-6) and C-reactive protein (CRP), PCT after early resuscitation in patients with severe acute pancreatitis

**DOI:** 10.5937/jomb0-55659

**Published:** 2025-08-21

**Authors:** Jinlong Wang, Zhanghua Guo, Xin Chai, Dupeng Li, Binxiao Su, Peng Jiang

**Affiliations:** 1 Xijing Hospital, Fourth Military Medical University, Department of Critical Care Medicine, Xi'an, Shaanxi Province, China

**Keywords:** serum interleukin-6 (IL-6), C-reactive protein (CRP), PCT, early fluid resuscitation rate, severe acute pancreatitis, inflammation, complications, serumski interleukin-6 (IL-6), C-reaktivni protein (CRP), PCT, rana stopa nadoknade tecnosti, teški akutni pankreatitis, zapaljenje, komplikacije

## Abstract

**Background:**

This study investigated the effects of different early resuscitation fluid replenishment rates (FRRs) on inflammation (serum interleukin-6 (IL-6) and C-reactive protein (CRP), PCT and complications in patients with severe acute pancreatitis (SAP).

**Methods:**

Sixty-six patients with SAP were studied. According to the ratio of total fluid replenishment 24 h after admission to 72 h (FRR), the patients were rolled into a low FRR group (Low group), a moderate FRR group (Moderate group), and a high FRR group (High group), with 22 cases in each. Serum-related indexes, APACHE II score, HCT, systemic inflammatory response syndrome (SIRS) duration, length of hospital stay (LOS), and complication rate (CR) were determined and compared.

**Results:**

The results suggested that ALT, AST, SCr, BUN, TBil, APACHE II, scores and HCT in the Moderate group were the lowest (P< 0.05), while those in the High group were the highest (P<0.05). After the patients were treated for 72 h, the IL-6, CRP and PCT in the Low and High groups were higher than those in the Moderate groups, exhibiting differences with P<0.05 and P<0.01, respectively. The SIRS duration and LOS in the Low and High groups were longer. They presented differences with P<0.05 and P<0.01 to the Moderate group, respectively. The rates of MODS, mechanical ventilation, pancreatic necrosis infection, and death in the Moderate group were the lowest (P<0.05).

**Conclusions:**

the moderate FRR could effectively alleviate the inflammatory response of patients with SAP shorten the treatment time, and reduce the CR.

## Abstract (sr)

**Uvod:**

Ova studija je istraživala efekte razlicitih ranih stopa nadoknade tecnosti (FRRs) na zapaljenje (serumski inter-leukin-6 (IL-6), C-reaktivni protein (c Rp), PCT i komp-likacije kod pacijenata sa teškim akutnim pankreatitisom (SAP).

**Metode:**

Ispitivano je šezdeset šest pacijenata sa SAP. Prema odnosu ukupne nadoknade tecnosti u periodu od 24 do 72 casa nakon prijema (FR R ), pacijenti su raspoređeni u grupu sa niskim FRR (Niska grupa), umereni FRR (Umerena grupa) i visoki FRR (Visoka grupa), sa po 22 pacijenta u svakoj grupi. Određivani su i upoređivani serumski pokazatelji, APACHE II skor, HCT, trajanje sistem-skog inflamatornog odgovora (SIRS), dužina boravka u bol-nici (LOS) i stopa komplikacija (CR).

## Introduction

Severe acute pancreatitis (SAP) is a common critical digestive disorder. It is characterised by rapid onset, rapid progression, multiple complications, complex treatment, and high fatality rate [1]. Its occurrence is related to overeating, alcoholism, biliary diseases, and other factors, with pancreatic necrosis, cytokine activation, systemic inflammatory response, multi-organ dysfunction syndrome and other major clinical features [2]. The clinical manifestations of SAP include cyanosis, abdominal hypertension, decreased urine volume, shortness of breath, and confusion [3]. Thanks to improving living standards these years, the risk of SAP has gradually increased [4]. According to statistics, the annual incidence of SAP worldwide is 13-45 cases/100,000, which is around 20% of all patients with acute pancreatitis, among which the SAP mortality rate is up to 15.6% [5]. Thanks to the research on the SAP mechanism and the development of medical technology, the case fatality rate of SAP has decreased by about 10%. However, its treatment cost and case fatality rate are still high [6]. Currently, the treatment methods used for SAP include surgery, symptomatic therapy, early fluid resuscitation therapy and systemic support therapy, among which surgical treatment has achieved significant curative effect by removing pancreatic necrotic tissue but increases the mortality rate of SAP patients [7]. Symptomatic treatment methods such as routine fasting, fluid replenishment, and antiinfection treatments still have limitations in alleviating the progression of SAP [8]. Fluid resuscitation is one of the main treatment methods to maintain the stability of body circulation and the metabolism of various organ systems [9].

SAP patients lose many body fluids, resulting in a sharp decrease in effective circulating blood volume and even hypovolemic shock, which eventually leads to death. Early fluid resuscitation therapy can correct hypovolemic shock in time, which is an important measure to prevent and cure hypovolemic shock and multiple organ function injury to improve the prognosis and cure rate of SAP patients. The controlled fluid resuscitation strategies have been confirmed to be pivotal in SAP treatment [10] [11]. Currently, there are two kinds of liquid resuscitation methods for SAP treatment in clinics: crystal and artificial colloidal. However, there is currently no unified clinical standard for the amount of fluid in early liquid resuscitation and the speed of fluid replenishment [12], which is still controversial. Therefore, this work aimed to take SAP patients as the research object to analyse the influence of different FRRS on the inflammation and complications of SAP patients to provide certain clinical guidance for selecting clinical treatment plans for SAP patients.

## Materials and methods

### Research objects

Sixty-six SAP patients who visited our hospital from January 2023 to October 2024 were enrolled, including 36 males and 30 females. The patients were 46.53±10.26 years old on average, ranging from 18 to 73 years. This work was approved by Xijing Hospital, Fourth Military Medical University Medical Ethics as a member. The included subjects and their families were all aware of the content of this work, signed informed consent, and agreed to participate.

The criteria for enrolling the patients in this work were described as follows: I. patients satisfying the SAP diagnostic criteria; II. patients over 18 years of age; III. Patients with the time from onset to hospitalisation were within 48 hours; IV patients who did not receive any medical intervention before admission; and V patients with chronic underlying diseases such as heart, liver, lung, kidney, and brain. The criteria for which the patients were excluded from this work were: I. patients with malignant tumours; II. patients with autoimmune diseases; III. patients with chronic heart failure and severe liver, kidney, and other organ diseases; IV patients with severe gastrointestinal dysfunction and a history of gastrointestinal or pancreatic surgery; V patients who are accompanied by mental illness or cognitive impairment; VI. pregnant and lactating women; and VII. patients with chronic pancreatitis.

### Grouping

Based on the difference between the total fluid replenishment ratio (FRR) at 24h and 72h after admission, the included objects were rolled randomly into a Low, a Moderate, and a High group, with 22 cases in each group. FRR of the Low group was less than 0.35, that in the Moderate group was 0.35 0.45, and that in the High group was >0.45. 13 males and 9 females were enrolled in the Low group, and they were 45.81±9.83 years old. In the Moderate group, 12 males and 10 females were 47.29±13.29 years old. The High group enrolled 11 males and 11 females, 46.01±10.03 years old. No significant differences were observed in gender proportion and mean age of patients in different groups receiving various FRRs (P>0.05).

### Treatment methods

All subjects were treated with fasting, water prohibition, gastrointestinal decompression, acid inhibition, enzyme inhibition, proton pump inhibitors, antiinfection, and other conventional treatments. Early liquid resuscitation was also administered with 0.9% sodium chloride solution (Jiangsu Hengrui Pharmaceutical Co., LTD.) and 6% hydroxyethyl starch solution (Fresenius Kabi Deutschland GmbH). Patients with kidney failure or more severe disease should also be given blood purification treatment; Patients with gastrointestinal or abdominal bleeding should be treated with digestive endoscopic hemostasis. Patients with hypotensive shock were given pressors to maintain blood pressure half an hour before fluid resuscitation. Patients in the Low, Moderate, and High groups were given different rates of fluid replenishment and were continued with 0.9% NaCl solution if the resuscitation goal was not achieved under different fluid resuscitation FRRs. Early liquid resuscitation to reach the following 2 points or more was considered to be the recovery standard: urine volume was 0.5 mL/(kg*h), heart rate was maintained at 80-110 beats/min, hematocrit (HCT) was 30%, central venous oxygen saturation was 70%, central venous pressure was maintained at 8-12 mmHg, and mean venous pressure was 65 mmHg.

### Observed indicators

- I. Age, sex ratio, aetiology, time from onset to medical treatment, acute physiology, Acute Physiology and Chronic Health Evaluation II (APACHE II) score, abdominal pressure, HCT, and other basic clinical data were collected.
- II. Serum was collected 72h after treatment, and the changes of alanine aminotransferase (ALT), aspartate aminotransferase (AST), urea nitrogen (BUN), serum creatinine (SCr), and total bilirubin (TBiL) were detected by automatic biochemical analyser (Hitachi 7600).
- III. The changes in APACHE II score and HCT in the three groups were analysed before treatment and after they were treated for 24h, 48h, and 72h.
- IV The levels of inflammatory response factors in the Low, Moderate, and High groups were compared before and 72h after treatment. Fasting venous blood was collected before and 72h after they were treated. The serum was separated, and the contents of interleukin-6 (IL-6) and C-reactive protein (CRP) were determined using an ELISA kit (Shanghai Aibo Biomedicine Technology Co., LTD.). The procalcitonin (PCT) content in serum was determined by immunoluminescence method [13].
- V. The duration of systemic inflammatory response syndrome (SIRS)
- VI. The post-treatment complications rate (CR) of patients was analysed, mainly including multiple organ dysfunction syndrome (MODS) [14], mechanical ventilation, pancreatic necrosis infection, and death.

### Methods for statistics

SPSS 22.0 was utilised for data analysis. The measurement data with normal distribution and homogeneity of variance were expressed as mean ± standard deviation (x̄±s). The differences between groups were analysed using a parameter test; otherwise, they were processed using the Mann-Whitney U test. The count data was expressed as percentages (%), and a chi-square test was adopted. The Kruskal-Wallis H test tested multiple independent samples, with P<0.05 indicating statistically significant differences.

## Results

### Comparison of aetiology and clinical indexes on admission of patients

The leading causes of SAP were bile, alcohol, and high fat. There were 6, 9, and 4 patients with bile, alcohol, and high-fat SAP in the Low group and 7, 7, and 6 patients in the Moderate group, respectively. In the High group, there were 8 patients with bile SAP, 7 patients with alcoholic SAP and 5 patients with high fat SAP. The proportion of patients with different etiologies exhibited no sharp differences, showing P>0.05. The time from onset to treatment, APACHE II score at admission, and HCT at admission in SAP patients with different FRRs exhibited no significant differences (P>0.05) (Table 1).

**Table 1:** Etiology and clinical indicators at the admission of patients in different groups.

	Low group (n = 22)	Moderate group (n = 22)	High group (n = 22)	*P*-value
Aetiology				0.126
Bile	6	7	8	
Alcohol	9	7	7	
High fat	4	6	5	
Other	3	2	2	
APACHE II score at admission	11.45 ± 5.61	12.06 ± 6.23	11.98 ± 5.04	0.163
HCT at admission (%)	41.8 ± 6.32	42.2 ± 5.25	41.5 ± 7.04	0.254
Time from onset to treatment (h)	30.27 ± 12.01	31.28 ± 10.78	29.98 ± 12.46	0.187

### Comparison of biochemical indexes of patients after 72h treatment

Biochemical markers of all SAP patients were compared, and the results were listed in Table 2. The ALT, AST, SCr, BUN, and TBil in the Moderate group were the lowest and exhibited great differences with those in the Low and High groups (*P*<0.05), while those in the Low group were much lower and presented obvious differences based on the High group (*P*<0.05).

**Table 2:** Biochemical indexes of patients from different groups after 72h treatment.

	Low group (n = 22)	Moderate group (n = 22)	High group (n = 22)	P-value
ALT (U/L)	238.56 ± 37.24	226.19 ± 30.37	259.19 ± 26.75	< 0.05
AST (U/L)	178.27 ± 23.66	152.74 ± 16.32	225.32 ± 19.54	< 0.05
SCr (μmol/L)	94.48 ± 14.97	92.26 ± 14.21	97.03 ± 12.37	< 0.05
BUN (mmol/L)	8.97 ± 3.36	6.62 ± 1.95	10.18 ± 2.35	< 0.05
TBil (μmol/L)	35.29 ± 3.42	28.57 ± 3.39	39.19 ± 4.04	< 0.05

### Analysis of changes in APACHE II score and HCT of patients at different time periods

**Figure 1:**
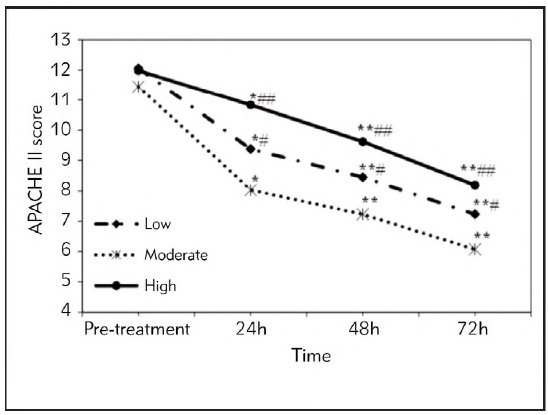
APACHE II scores of SAP patients in different time periods from different groups.<br>(* and ** meant a difference with *P*<0.05 and *P*<0.01 to the score before the patients were treated, respectively; # and ## indicated a difference with *P*<0.05 and *P*<0.01 based on that in the Moderate group, respectively.)

Before treatment (at admission), the APACHE II scores among SAP patients in the Low, Moderate, and High groups showed no observable differences (*P*>0.05). APACHE II scores of the SAP patients with different FRRs in various groups showed an obvious downward trend after early liquid resuscitation and rehydration treatment. Among them, the APACHE II scores in the Moderate group showed the most significant decline. At 24h after treatment, the APACHE II scores of SAP patients in all groups were remarkably different from those before they were treated (*P*<0.05), and that in the Moderate group was greatly lower based on the scores in the High group (*P*<0.01) and the Low group (*P*<0.05). After the SAP patients were treated for 48 and 72 hours, the APACHE II scores of all patients were remarkably lower than before they were treated (*P*<0.01), and the APACHE II score in the Moderate group was much lower based on the values in the High group (*P*<0.01) and the Low group (*P*<0.05). The above results were demonstrated in Figure 1.

**Figure 2:**
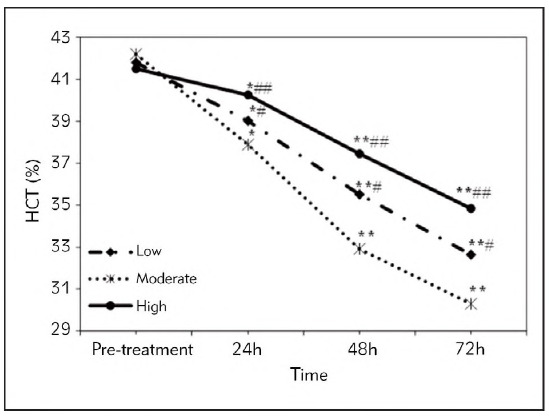
HCT values of SAP patients in different periods from different groups.<br>(* and ** meant a difference between P<0.05 and P<0.01 to the score before the patients were treated with different FRRs, respectively; # and ## indicated a difference with P<0.05 and P<0.01 based on the score in the Moderate group, respectively)

It was observed no visible difference in HCT values of SAP patients in the Low, Moderate, and High groups before they were treated (*P*>0.05). The HCT values of all SAP patients showed an obvious decreasing trend after early liquid resuscitation and replenishment treatment, and that in the Moderate group exhibited the most significant decreasing trend. At 24h after treatment, the HCT values of SAP patients in the Low, Moderate, and High groups were statistically different from those at admission (*P*<0.05), and that in the Moderate group was sharply lower based on the value in the High group (*P*<0.01) and the Low group (*P*<0.05). After 48h and 72h upon the patients were treated, the HCT values in SAP patients in the Low, Moderate, and High groups were all decreased, and the differences to those at admission exhibited significance with *P*<0.01; that in the Moderate group was greatly lower based on the value in the High and Low groups, exhibiting differences with *P*<0.01 and *P*<0.05, respectively (Figure 2).

### Changes in inflammatory factors of patients before and after they were treated

**Figure 3:**
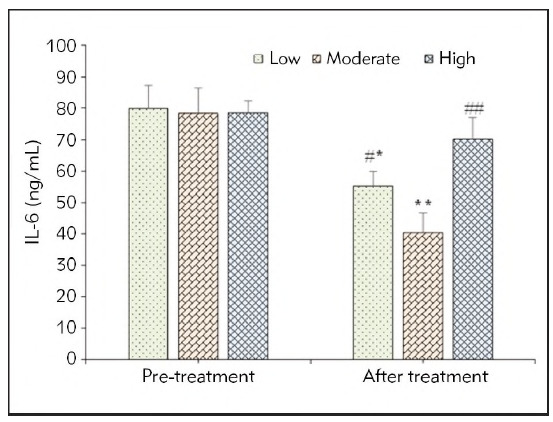
IL-6 values of SAP patients in different periods from different groups.<br>(* and ** meant a difference with P<0.05 and P<0.01 to the score when they were treated at different FRRs, respectively; # and ## indicated a difference with P<0.05 and P<0.01 based on the score in the Moderate group, respectively)

No obvious difference was observed in IL-6 values of all SAP patients in different groups before they were treated (*P*>0.05). 72h later when the patients were treated, IL-6 was decreased when the FRR of patients was low (*P*<0.05), greatly decreased at the moderate FRR (*P*<0.01), and exhibited no great difference at the high FRR (*P*>0.05) in contrast to the values before they were treated. In addition, IL-6 of patients with moderate FRR was lower based on that in patients with low FRR (*P*<0.05) and with high FRR (*P*<0.01). Figure 3 displayed the specific variations in inflammatory factors of patients when they were treated at different FRRs.

**Figure 4:**
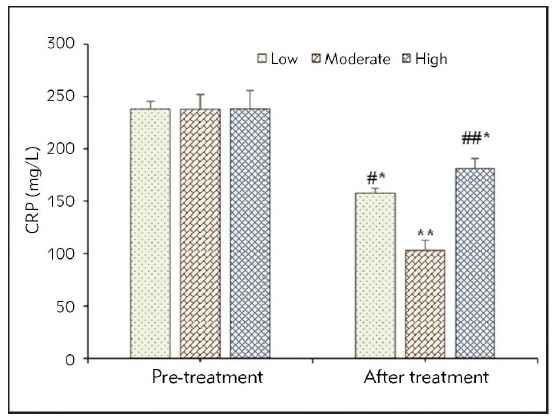
CRP values of SAP patients in different periods from different groups.<br>(* and ** meant a difference between P<0.05 and P<0.01 to the values before the patients were treated, respectively; # and ## indicated a difference with P<0.05 and P<0.01 based on the value of patients treated at moderate FRR, respectively)

No remarkable difference was found in CRP values of SAP patients before they were treated no matter which group they were enrolled in (*P*>0.05). After the patients were treated 72h, the CRP values in patients receiving the low and high FRRs were decreased (*P*<0.05), while that in patients with the moderate FRR was decreased with a significance of *P*<0.01. CRP value in patients with moderate FRR was lower based on that in those at the low FRR (*P*<0.05) and high FRR (*P*<0.01). Figure 4 illustrated the detailed data for above results.

**Figure 5:**
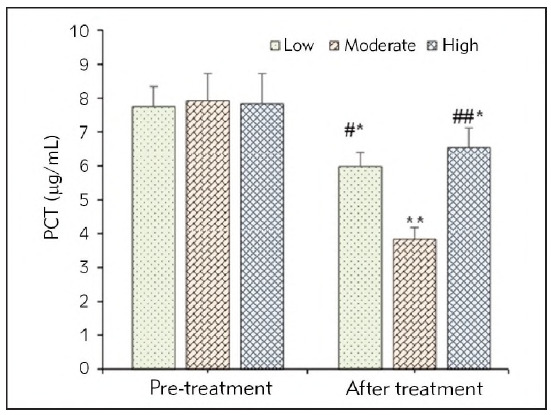
PCT values of SAP patients in different periods from different groups.<br>(* and ** meant a difference between P<0.05 and P<0.01 to the values before the patients were treated, respectively; # and ## indicated a difference with P<0.05 and P<0.01 based on the value for patients treated at moderate FRR, respectively)

No remarkable difference was observed in PCT values of SAP patients before they were treated no matter which group they were enrolled in (*P*>0.05). 72h after the patients were treated, the PCT values at the low and high FRRs were decreased (*P*<0.05), while that in patients treated with moderate FRR was decreased with a significance of *P*<0.01. The PCT value in the Moderate group was lower based on that in patients with the low FrR (*P*<0.05) and high FRR (*P*<0.01). Figure 5 illustrated the detailed data for above results.

### Comparison of SIRS duration and LOS of patients with different FRRs

**Figure 6:**
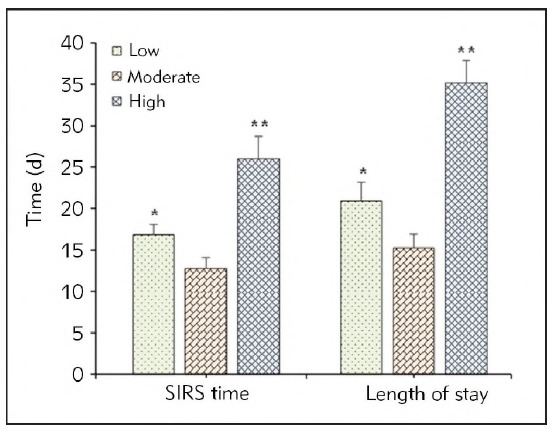
Comparison of SIRS duration and LOS among SAP patients from various groups.<br>(* and ** meant a difference with P<0.05 and P<0.01 based on the values in the Moderate group, respectively.)

The SIRS duration and LOS of patients in the Low group were 16.85 ± 1.25 and 20.92 ± 2.23, respectively, while those in the High group were 26.01 ± 2.69 and 35.17 ± 2.67, respectively; while those in the Moderate group were 12.74 ± 1.31 and 15.26 ± 1.56, respectively. The SIRS duration and LOS in the Moderate group were the shortest and exhibited a great difference with *P*<0.05 and *P*<0.01 to the patients who were treated at low and high FRRs, respectively. Figure 6 demonstrated the detailed information of above results.

### The CR of patients after treatment after they were treated at different FRRs

**Figure 7:**
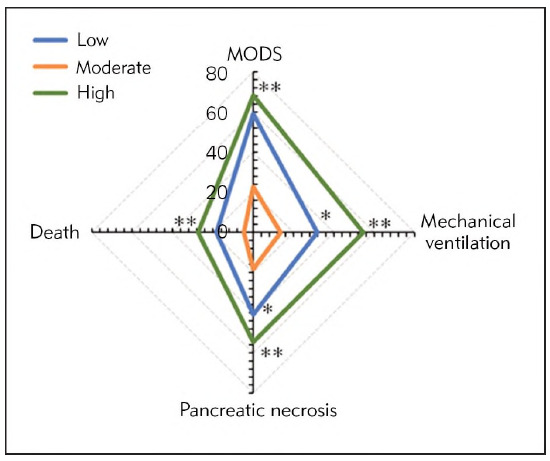
Comparison of CR of patients from different groups with various FRRs.<br>(* and ** meant a difference with P<0.05 and P<0.01 based on the values in the Moderate group, respectively)

The incidences of MODS after the SAP patients in the Low, Moderate, and High groups were treated were 13 (59.09%), 5 (22.73%), and 15 (68.18%), respectively. Thus, the patients treated at moderate FRR exhibited the lowest incidence of MODS among all groups (*P*<0.01). The rate of mechanical ventilation after the SAP patients were treated were 7 (31.82%) VS 3 (13.64%) VS 12 (54.55%), respectively, no matter which groups they were from. It suggested that the rate of mechanical ventilation in the Moderate group was the lowest and exhibited great differences with *P*<0.01, and that in the Low and High groups also showed a difference with *P*<0.05. The numbers of patients with pancreatic necrosis infection in SAP patients who were treated at low FRR (9 cases, 40.91%) and high FRR (12 cases, 54.55%) were more than that in the Moderate group (4 cases, 18.18%), showing sharp differences with *P*<0.01. The numbers of patients with post-treatment death who were treated at low, moderate, and high FRRs were 4 (18.18%) VS 1 (4.55%) VS 6 (27.27%), respectively. Therefore, patients treated at moderate FRR exhibited a visibly lower incidence of death based on that in patients with low FRR (*P*<0.01) and high FRR (*P*<0.05). The above results were illustrated in Figure 7 below.

## Discussion

SAP will cause the body's immune system to release a large number of inflammatory factors, leading to SIRS, resulting in multiple organ function impairment in patients, and eventually lead to death. Current research results [15] [16] show that SAP patients have two peak periods of death. The first period is the first week of SIRS, and about 50% of patients die from MODS. In the second stage, one week after the occurrence of SIRS, most patients died due to complications such as infection. Therefore, early liquid resuscitation fluid replenishment is conductive to treating and prognosing the SAP patients. However, there is no unified standard on the dosage and rate of early liquid resuscitation fluid replenishment. Therefore, SAP patients were based in this work to analyse the influence of FRR on the inflammation and complications of SAP patients. It was found that ALT, AST, SCr, BUN, TBil, APACHE II scores, and HCT in the Moderate group were lower than those in patients treated at the low and high FRRs (*P*<0.05). It is suggested that different rates of liquid resuscitation rehydration can improve patients' biochemical indexes, and different rates of liquid resuscitation rehydration can improve patients' kidney and lung function to varying degrees. The Moderate group showed a more significant decline in biochemical indices and was more advantageous in SAP treatment. Fluid resuscitation fluid can relieve blood volume loss, improve blood concentration, and effectively reduce SAP mortality [17] [18]. Rapid fluid resuscitation can't effectively improve the ischemic and anoxic state of SAP patients, and will lead to the rapid reduction of HCT, as well as the occurrence of complications such as acute pulmonary edema, brain edema and systemic soft tissue edema [19] [20]. At the same time, studies have shown that rapid fluid resuscitation can lead to a decrease in oxygen content in tissue cells, and thus an increase in APACHE II scores of patients [21]. Therefore, the APACHE II score of SAP patients treated at the high FRR was observably higher based on those in patients treated at moderate and low FRRs, which kept in line with the results of the present study. In addition, it was observed that after treatment, the CRP IL-6, and PCT decreased in the Low, Moderate, and High groups compared with those before the treatment, with the most significant decrease in the Moderate group. It is suggested that FRR can better relieve the inflammatory response in SAP patients, which may be due to the matching between the degree of capillary leakage and moderate FRR [22]. It can better improve the microenvironmental circulation state of the body, and then control the expansion of inflammatory response, and finally promote the decrease of the content of inflammatory factors. Finally, the SIRR duration and LOS of patients who were treated at moderate FRR were greatly shorter and exhibited obvious difference with *P*<0.05 TO those in patients treated at low and high FRRs. The incidences of MODS, mechanical ventilation, pancreatic necrosis infection, and death were much lower than those in the High and Low groups (*P*<0.05). It is suggested that the moderate FRR shortens the SIRS duration and LOS, and reduces post-treatment CR in SAP patients. Due to various factors, release of inflammatory factors in the body of SAP patients increases, and the hemodynamic stability is poor [23] [24]. Rapid fluid replenishment aggravates the load of various tissues and organs in SAP patients and promotes the deterioration or progression of the disease [25] [26]. The moderate FRR may have an advantage in maintaining blood stability, thus normalising the oxygen content of tissues and organs, while effectively controlling the inflammatory response and reducing the duration of treatment.

## Conclusion

This work investigated the effects of different FRRS in early fluid resuscitation on inflammation and complications in SAP patients. The results revealed that the early medium speed fluid resuscitation could effectively improve the inflammatory response of SAP patients, shorten the treatment time, and reduce CR, which played a positive role in SAP treatment. However, this work was subjected to several shortcomings. The number of SAP patients enrolled and the clinical indicators collected were limited, and there may be some deviations in the study results. In future work, the sample size will be further expanded to verify and analyse the results of this work. In conclusion, this work offered a certain clinical basis for the selection of clinical treatment for SAP patients.

## Dodatak

### Acknowledgments

We would like to express our gratitude to the Department of Critical Care Medicine, Xijing Hospital, Fourth Military Medical University, for their support during this study. Special thanks to the patients and their families for their participation and trust.

### Funding

This study received no funding.

### Ethical considerations

This study was approved by the ethics committee of Xijing Hospital, Fourth Military Medical University. Written informed consent was obtained from all participants prior to their inclusion in the study.

### Author contributions

Jinlong Wang and Zhanghua Guo conceptualised the study. Xin Chai and Dupeng Li performed the experiments. Binxiao Su and Peng Jiang analysed the data. All authors contributed to drafting and revising the manuscript and approved the final version for submission.

### Conflict of interest statement

All the authors declare that they have no conflict of interest in this work.
